# Fast Decay of CaMKII FRET Sensor Signal in Spines after LTP Induction Is Not Due to Its Dephosphorylation

**DOI:** 10.1371/journal.pone.0130457

**Published:** 2015-06-18

**Authors:** Nikolai Otmakhov, Shaurav Regmi, John E. Lisman

**Affiliations:** Biology department, Brandeis University, Waltham, MA 02454, United States of America; Louisiana State University Health Sciences Center, UNITED STATES

## Abstract

Because CaMKII is the critical Ca^2+^ sensor that triggers long-term potentiation (LTP), understanding its activation and deactivation is important. A major advance has been the development of a FRET indicator of the conformational state of CaMKII called Camui. Experiments using Camui have demonstrated that the open (active) conformation increases during LTP induction and then decays in tens of seconds, with the major fast component decaying with a time-constant of ~ 6 sec (tau1). Because this decay is faster if autophosphorylation of T286 is prevented (the autophosphorylation prolongs activity by making the enzyme active even after Ca^2+^ falls), it seemed likely that the fast decay is due to the T286 dephosphorylation. To test this interpretation, we studied the effect of phosphatase inhibitors on the single-spine Camui signal evoked by two-photon glutamate uncaging. We applied inhibitors of PP1 and PP2A, two phosphatases that are present at synapses and that have been shown to dephosphorylate CaMKII *in vitro*. The inhibitors increased the basal Camui activation state, indicating their effectiveness in cells. However, in no case did we find that tau1 was prolonged, contrary to what would be expected if the decay was phosphatase-dependent. This could either mean that decay was due to some unknown phosphatase or that the decay was not due to dephosphorylation. To distinguish between these possibilities, we expressed pseudo-phosphorylated Camui (T286D) (plus additional mutations [T/A] that prevented inhibitory 305/306 phosphorylation). This form had an elevated basal activation state, but was further activated during glutamate uncaging; importantly the activation state decayed with tau1 nearly the same as that of WT Camui. Therefore, the data strongly indicate that tau1 is not due to T286 dephosphorylation. We conclude that, although Camui is an excellent tool for observing CaMKII signaling, further experimentation is needed to determine how CaMKII is turned off by its dephosphorylation.

## Introduction

Long-term potentiation (LTP) occurs during learning and is thought to underlie memory [1, 2]. CaMKII is activated by the elevation of intracellular Ca^2+^ that occurs during LTP induction, an activation that is necessary to produce potentiation of AMPAR-mediated transmission [3–5]. Other recent work suggests that CaMKII, once bound to the NMDAR, has a role in the maintenance of LTP, suggesting that there may be long-lasting changes in the state of CaMKII [6, 7]. The biochemistry of CaMKII has demonstrated that there are mechanisms for producing persistent activation of CaMKII after Ca^2+^ levels fall. Notably, Ca^2+^ stimulates autophosphorylation of T286; once this occurs, the enzyme stays partially active (autonomous) even after Ca^2+^ levels fall [8, 9]. Importantly, when T286 is mutated so that phosphorylation of this site cannot occur, LTP is greatly reduced and there are devastating effects on memory [10, 11].

Given this role of CaMKII in memory processes, it is critical to have experimental methods for measuring CaMKII activation at spines. Early work demonstrated that LTP induction produces a persistent increase in CaMKII autophosphorylation [12, 13], but this work measured the state of CaMKII in hippocampal homogenates and so did not give specific information about the pool of CaMKII at synapses. It was thus a major step forward when an optical indicator of CaMKII, Camui, was developed [14, 15]. CaMKII has 12 catalytic subunits held together by a central hub. The Camui indicator is based on Forster Resonance Energy Transfer (FRET) between two fluorophores fused to the catalytic (N terminus) and association (C terminus) domains of CaMKIIα subunits. Since ~12 CaMKII subunits are combined by their association domains to the central hub of the holoenzyme the change in FRET provides a measure of the displacement of the regulatory and catalytic region of CaMKII from the central hub. In the inactive state, the pseudo-substrate region of the regulatory loop lies in the catalytic site and inhibits enzyme activity. The binding of Ca^2+^-associated calmodulin (CaM) to the regulatory loop moves the regulatory and catalytic regions away from the hub and displaces the regulatory region from the catalytic region, thereby allowing catalytic action [16]. Thus, at least in a crude way, the position of the catalytic region, as measured by FRET, is related to the activity of the enzyme. Direct comparison of the FRET signal of Camui with its enzymatic activity is consistent with this conclusion [14, 15, 17].

Several major conclusions were derived from the study of Camui signals during LTP induction at individual CA1 hippocampal spines [14, 18]. First, it was found that CaMKII activation, as measured by the magnitude of the FRET change, was rapid (reaching a plateau within ~ 6 sec) during LTP induction (repetitive glutamate uncaging at 0.5 Hz). Second, the activation was specific to the stimulated spine, demonstrating that the localization of activated CaMKII is sufficient to account for the synapse specificity of LTP [19, 20]. Third, both the peak and duration of the activation of CaMKII were enhanced by autophosphorylation of T286, as expected from previous biochemical work [8, 9, 21, 22]. However, the prolongation of the major deactivation time-constant (tau1) as a result of autophosphorylation was surprisingly short: from ~2 sec for 286A to ~5–9 sec for WT Camui, suggesting that the major action of CaMKII might be brief because of rapid dephosphorylation.

Understanding the dynamic of the autophosphorylated CaMKII is of particular importance, given the essential role of T286 phosphorylation in LTP induction and its proposed role in LTP maintenance [3, 7, 11, 23]. We therefore sought to understand the phosphatase reactions suspected of producing the decay of the Camui signal. Unexpectedly, however, our results demonstrate that the rapid decay of the Camui signal is not due to dephosphorylation.

## Materials and Methods

### Slice culture preparation and DNA transfection

Hippocampal slice cultures were prepared from Long-Evans rats at P6–9 and were maintained for 11–14 days before cDNA transfection as described earlier [24]. WT and 286A mutant of Camui cDNA [14] were a kind gift of Ryohei Yasuda (Florida Max Plank Institute). cDNAs of mCherry fused protein phosphatase inhibitors of PP1(NM_138689.2) and PP2A (NM_003011.3) and their less active variants were made by David Brautigan (University of Virginia School of Medicine) as part of the grant subcontract. These were PP1 inhibitors: mCherry-Phi1(T57A) and mCherry-Phi1 (T57D) and PP2A inhibitors: mCherry-SET(S9A/S93A) and mCherry-SET (S9D/S93D). Both Phi1 (A and D variants) and SET (A and D variants) are very potent (with Ki in nM range) and specific inhibitors of PP1 and PP2A respectively (see more description in S2 Text). T286D/T305A/T306A Camui mutant was constructed by cutting with XcmI and EcoNI and pasting using GFPCaMKII-T286D/T305A/T306A [10] and WT Camui as source vectors. The Camui T286D/T305D/T306D and T286A/T305A/T306A were constructed by cutting with the same enzymes and pasting using Camui T286D and Camui T305D/T306D or Camui T286A and T305/T306A as vector sources respectively. The Camui T305/T306D construct was prepared by cutting with the same enzymes and pasting using GFP-CaMKII T305D/T306D [10] and Camui WT as vector sources. GFP-CaMKII T305D/T306D was a kind gift of Paul De Koninck.

The presence of mutations was confirmed by sequencing. The study was carried out in strict accordance with the recommendations in the Guide for the Care and Use of Laboratory Animals of the National Institutes of Health. The protocol was approved by the Committee on the Ethics of Animal Experiments of the Brandeis University (PHS Animal Assurance #: A3445-01; Protocol #13001)

### Spine stimulation and imaging

One or two days after electroporation, slices expressing Camui were cut out of the 6-well insert membranes used for their support and were transferred to the glass bottom of a custom-made imaging chamber placed on a custom-made motorized stage. Slices were completely submerged in a circulating ACSF (flow rate 2.5–3 ml/min) of the following composition (in mM): 124 NaCl, 2.5 KCl, 4 CaCl_2_, 0 MgCl_2_, 1.25 NaH_2_PO_4_, 26 NaHCO_3_, 20 Dextrose, 0.001 TTX, balanced with 95% O_2_ and 5% CO_2_, pH 7.4 at room temperature (22C°–24C°). Caged MNI-glutamate (at final concentration of 4 mM) was added to the bath ACSF after 10–15 min of slice incubation to prevent possible slice excitation. Slices were equilibrated in these conditions for at least 20 min before imaging. Imaging was performed using a custom-made two-photon microscope system, as described in [14]. The system was equipped with two Coherent two-photon IR lasers (one for imaging and one for uncaging), two fast PMTs (H7422-40), SPC150 FLIM board (Becker- Hickl, DE), 2 Pockels cells (Conoptics, CT), and 60× 1.0 NA WI (Olympus, JA) objective. Time-lapse fluorescent lifetime imaging (FLIM) and analysis were performed using custom software, as described in [14]. GFP and mCherry were excited by mode-locked IR light at 920 nm. Green and red emissions were separated by DCXR565 nm dichroic and HQ510/70 nm and HQ630/60 blocking filters (Chroma). Weak laser intensity (~1.0 mW under the objective) was used to minimize bleaching and photodamage. Green PMT signal was recorded in single-photon counting mode (SPC), and two types of images were constructed: one presenting averaged number of photons per pixel (SPC image) and the second presenting averaged single photon lifetime per pixel (FLIM). Red images were taken in standard integral mode. Single focal plane two channel images (64 × 64 pixel at 0.05 μm pixel size) were averaged online sequentially (24 images) and spatially (4 × 4 pixels), rendering final time-lapse images every 8 sec. Background was subtracted to determine cell edges, and ROIs were drawn around stimulated spine and nearby dendritic segment. Camui spine size was calculated as described in [25]. Spine activation was performed by local glutamate uncaging at 720 nm. The light spot was positioned at the distal edge of a spine head, and eight repetitive 6 msec pulses were delivered every 2 sec.

### Drugs

Stock solutions of Calyculin A (1 mM, LC Labs), and FK506 (40 mM, Invivogen) were prepared in DMSO. The final concentration of DMSO in ACSF was less than 0.1%. Stock solutions of caged MNI-glutamate (80 mM, Tocris), D, L-APV (20 mM, Sigma), and TTX (2 mM, Abcom Biochemicals) were prepared in deionized water. All stocks were kept frozen at −80C0° in aliquots until use.

### Statistical analysis

All data were presented as mean ± SE. Student’s t-test (Excel, 2010) was used to calculate statistical significance between sets of data.

## Results

**Fig 1:**
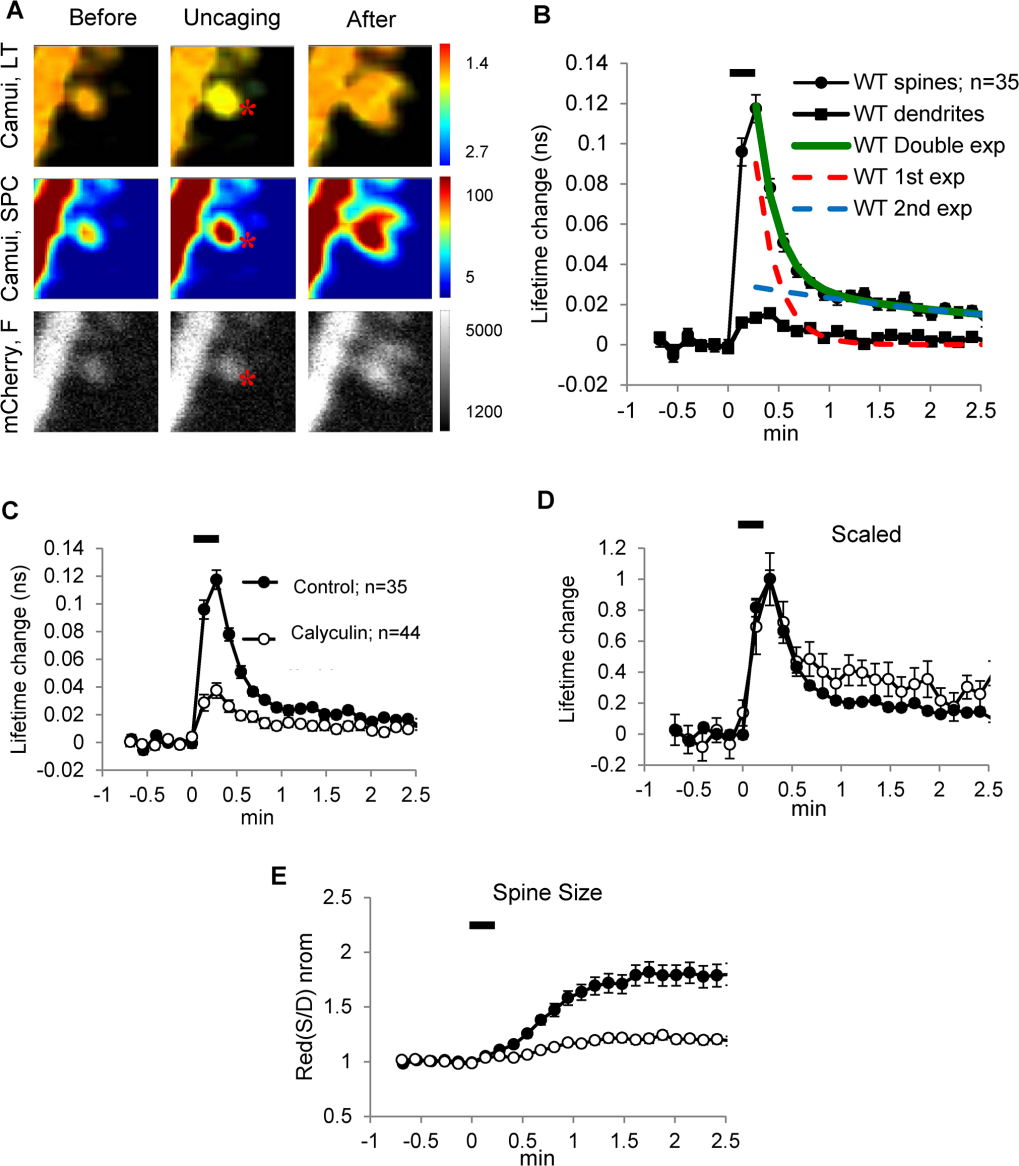
Fast decay of Camui signal is not affected by Calyculin A, an inhibitor of PP1/PP2A. (A) Top panel: lifetime images before, during, and after uncaging showing that the lifetime change of Camui (Camui, LT) is restricted to the stimulated spine, as indicated by the change of pseudo-color from orange to yellow; the location of glutamate uncaging is indicated by an asterisk. Middle panel: Camui content of spines, as measured by single-photon counting of GFP fluorescence (Camui, SPC), dramatically increased in stimulated spine. Bottom panel: fluorescent images of the volume marker mCherry (Cherry, F) showing spine enlargement after glutamate uncaging. Scale bar units: top–ns/pixel, middle–photons/pixel, bottom–AU. (B) Fluorescence lifetime response of WT Camui produced by glutamate uncaging (average of 35 spine experiments, filled black circles) overlapped with fitted double exponential (green) and underlying the first (dash red) and the second (dash blue) exponentials; dendritic response–black squires. (C–E) Graphs showing effects of Calyculin A (open symbols) on WT Camui fluorescence lifetime (raw, C and scaled, D) and spine size (E) in comparison to control conditions (filled symbols). Glutamate uncaging protocol (eight pulses at 0.5 Hz) was started at time 0 (horizontal black bar).

Fig 1 (black symbols) replicates previous results on the time course of the FRET lifetime signal during LTP induction [14]. Similar to this previous work, our experiments were done on cultured slices in which several pyramidal cells of CA1 had been transfected (1–2 days previously) with Camui. Camui is a FRET probe composed of CaMKIIα molecule fused with dVenus and GFP fluorophores on its N and C terminals, respectively. Upon activation, CaMKII changes from a closed to an open conformation, leading to a decrease in the FRET between the fluorophores. As described previously [14], FRET interactions can be monitored using the fluorescence lifetime of GFP; the transition from the close to the open state leads to an increase in the measured lifetime (see Methods for details). To induce LTP at a single identified spine, a submicron spot of light generated by a two-photon laser was positioned just distal to the spine (Fig 1A). The bath ACSF contained 0 Mg^2+^ so that activation of NMDARs would lead to channel opening even without substantial postsynaptic depolarization. Spine activation was induced by eight repetitive fast flashes (6 msec at 0.5 Hz) that locally converted the inactive caged MNI-glutamate present in the bath to active glutamate. As is standard with this method, the ability of repetitive uncaging flashes to induce functional synaptic plasticity was monitored by observation of a structural correlate, the enlargement of the dendritic spine at which glutamate was uncaged (Fig 1A and 1E). As previously reported, this enlargement develops within about 1 minute after the end of stimulation and is specific to the stimulated spine. This induction protocol produces activation of Camui, as indicated by the increase in the fluorescence lifetime of the FRET donor (Fig 1 A and 1B) [14]. After the end of stimulation, the lifetime undergoes recovery with two exponentials: the major “fast” component decayed with a tau1 of ~6 sec (tau1 = 6.6 ± 0.7 sec, A1 = 64 ± 3%, n = 35; see S1 Text and S1 Fig for additional data), the smaller “slow” component decayed with a tau2 of more than 1 min (tau2 = 92 ± 24 sec, A2 = 36 ± 3%). All of these observations closely match those previously reported [14]. Importantly, [14] found that a Camui mutant deficient for T286 autophosphorylation (T286A) showed a much smaller magnitude of response to repetitive glutamate uncaging and substantially shorter fast decay (tau1) than that of WT Camui. We have reproduced these results and confirmed that the magnitude of Camui (T286A) response was less than half of that of WT and that the fast decay of the response was more than twice shorter (tau1 = 2.1 sec, n = 45) than that in WT Camui (S1 Fig).

**Fig 2:**
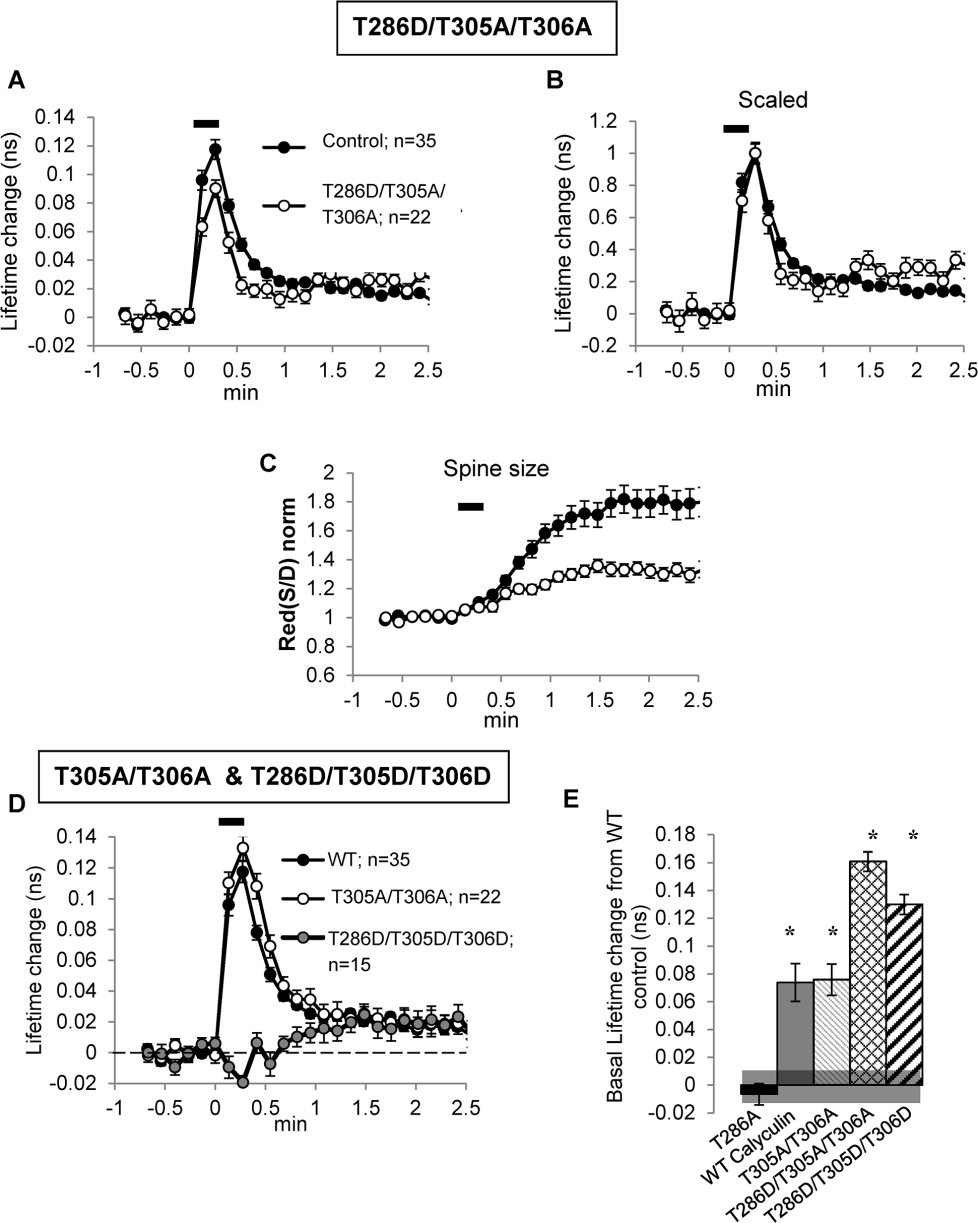
T286D/T305A/T306A Camui mutant is further activated by spine stimulation and has deactivation similar to that of WT Camui. (A, B, and D) Graphs of fluorescent lifetime change after glutamate uncaging of WT Camui (filled symbols), T286D/T305A/T306A and T305A/T306A Camui mutants (open symbols), T286D/T305D/T306D—gray symbols; (A) and (D), raw and (B), scaled data. (C) Change in spine size. Glutamate uncaging protocol (eight pulses at 0.5 Hz, horizontal black bar) started at time 0. (E) Bar diagram of basal fluorescence lifetime change in different experimental conditions in comparison to basal lifetime of WT Camui. Shadow line at the bottom indicates SE of basal lifetime for WT Camui. Stars indicate statistical significance change relative the basal lifetime of WT Camui.

On the hypothesis that the decay of the Camui signal reflects dephosphorylation, we expected that tau1 would be slowed by Calyculin A, a phosphatase inhibitor that affects both PP1 and PP2A, the two phosphatases implicated by biochemical analysis of hippocampal homogenates in the dephosphorylation of CaMKII [26]. Calyculin A (0.2–1 μM) was bath applied for 30–60 min before experiments and was retained during the experiments. Because all concentrations produced roughly similar results, the data were pooled together. As shown in Fig 1 (white symbols), the inhibitor had several dramatic effects. First, it increased the lifetime of the Camui signal under basal conditions by 0.076 ns (n = 44, p < 0.01, Fig 2E). Second, it reduced the magnitude of the lifetime change produced by the standard glutamate uncaging protocol ~ 3 fold (Fig 1C). Third, it reduced the increase of spine size produced by uncaging ~ 4 fold (Fig 1F). However, surprisingly, the fast component of decay was unchanged by phosphatase inhibition (tau1 = 5.9 ± 0.9 sec, p > 0.05). This can be most clearly seen when the responses were scaled to their peaks (Fig 1D). What Calyculin A did change about the decline of the response was the relative magnitude of the slow component measured at the end of the recording period (1.5–2.5 min after uncaging) (Fig 1E); its magnitude was approximately doubled by Calyculin A (29 ± 6% versus 15 ± 2%, p < 0.05).

The lack of effect of Calyculin A on the fast component was surprising, and we therefore tried several other phosphatase inhibitors. These included the PP2B inhibitor, FK506 and four expressible peptide inhibitors: two inhibitors of PP2A, SET(S9A/S93A) and SET(S9D/S93D) and two inhibitors of PP1, Phi1(T57A) and Phi1 (T57D). “D” substituted variants of both SET and Phi1 protein inhibitors are more potent than their “A” substituted variants (see details in S2 Text). The results of these experiments are presented in the supplementary material (S2 and S3 Figs and S2 Text). The main finding is that, although the inhibitors produced an increase in the basal lifetime, indicating their effectiveness in cells, in no case did we observe a slowing of the fast component.

The inability to affect the fast component might be because dephosphorylation of CaMKII is due to a phosphatase not affected by the inhibitors used. Indeed, there have been reports of a unique phosphatase of the PPM family that is specific to CaMKII [27, 28]. Alternatively, the fast component may *require* the phosphorylated state of T286 (if this phosphorylation is prevented by mutation [T/A], the decay is much faster [14]) but *not* be due to its dephosphorylation. To distinguish between these possibilities, we transfected neurons with Camui pseudophosphorylated at T286 (T286D/T305A/T306A; the T305/T306 sites were made nonphosphorylatable to prevent inhibitory phosphorylation [29]). If the FRET signal mainly depends on CaMKII phosphorylation and subsequent dephosphorylation at T286, this version of Camui should show little lifetime change during LTP induction. Fig 2A (white symbols) shows that, to the contrary, this Camui mutant was strongly activated by uncaging. The peak of the lifetime change in this mutant was only slightly smaller than that of WT Camui, which is surprising considering that its basal fluorescence lifetime was already significantly larger than that in WT Camui (increase 0.161 ± 0.01 ns, p < 0.05, Fig 2E, n = 22). Most importantly, the rapid lifetime decay, tau1, had kinetics similar to that of WT (Fig 2A and 2B). In fact, the fast decay (4.8 ± 0.5 sec, p < 0.05) was slightly but significantly faster than that of WT Camui. As can been seen in Fig 2A, the slow component of decay was also present in the T286D/T305A/T306A mutant. Indeed, the amplitude of this late slow component measured as the averaged amplitude at the end of recording period (1.5–2.5 min) was significantly higher (29 ± 3%, p < 0.05, Fig 2B) in the mutant relative to the WT control. This was also surprising and indicates that the decay of this component is not simply related to T286 dephosphorylation (see Discussion). As a control for the fact that the T286D mutant also had the T305A/T306A mutations, we used a Camui T305A/T306A mutant but with intact T286 site. With this form of Camui, the peak magnitude of the activation, as well as the fast (tau1) and slow (tau2) deactivation components, were very similar to those of WT Camui (tau1 = 8.3 ± 2 sec, n = 22; tau2 = 126 ± 64 sec; p > 0.05 for both, Fig 2D). The averaged amplitude of the slow component measured between 1.5 and 2.5 min was also not different from that of WT Camui. These data indicate that T305/T306 phosphorylation was not significantly involved in the activation or deactivation of Camui produced by glutamate uncaging and that the response properties of the triple mutant (T286D/T305A/T306A) described above were not due to the T305A/T306A mutations. These (T305A/T306A) mutations, however, did produce a significant elevation of the basal lifetime compared to that of WT Camui (0.076 ± 0.01 ns, p < 0.05, Fig 2E, n = 22), indicating that the inhibitory phosphorylation of T305/T306 restricts WT Camui activation at basal conditions. To confirm that Camui lifetime response to uncaging required the binding of CaM to CaMKII, as expected, we tested the T286D/T305D/T306D Camui mutant. This form of Camui imitates T286 phosphorylation but is unable to bind CaM. As expected this mutation produced elevation of the basal lifetime relatively to that of WT Camui (increase 0.130 ± 0.01 ns, n = 15, p < 0.001), but the fast response to glutamate uncaging was completely blocked (Fig 2D, gray symbols). Interestingly, the late component (measured at 1.5–2.5 min) of the response was still present and was no different from that of WT Camui (P > 0.05). Taken together, our results with Camui mutants clearly exclude the possibility that the fast component of the fluorescence lifetime decay of WT Camui (tau1) is a simple reporter of the dephosphorylation of T286. The results also suggest that the second component (tau2) has a complex nature and its decay does not simply reflect T286 dephosphorylation.

Another aspect of the results in Fig 2 is worthy of note. Since previous work demonstrated that expression of the T286D form of CaMKII produced an increase in spine volume [30], little further effect on spine volume by uncaging is expected. Fig 2C shows this to be the case.

## Discussion

Given the importance of CaMKII in LTP, the ability to monitor the activation state of the enzyme with an optical probe (Camui) is a major advance [14, 15, 18]. Understanding the Camui signal, however, requires a precise mapping of the conformational signal reported by the probe onto biochemically defined activation states. Previous work demonstrated a relationships of the Camui FRET signals to the biochemical states of CaMKII under *in vitro* conditions [14, 15, 17, 31]. Based on this data, initial studies on the spines of living cells showed that the Camui signal after LTP induction was very short-lived—less than a minute—such that the major (~80%) component decayed with tau1 of ~5–6 sec [14, 18]. Because this component was even faster if the T286 could not become phosphorylated (i.e., using T286A; results confirmed in the current study), it could sensibly be suspected that the decay reflects the dephosphorylation of T286, implying that the autonomous state of CaMKII is very short-lived because of rapid dephosphorylation. Our current results, however, are inconsistent with this interpretation. We found that the decay was not affected by phosphatase inhibitors. Most convincingly, the T286D pseudophosphorylated mutant of Camui, in which dephosphorylation cannot occur, still underwent strong activation and deactivation.

Several lines of evidence support the alternative interpretation that the fast component of decay of the Camui signal may be due to the loss of CaM “trapped” as a result of T286 autophosphorylation rather than the dephosphorylation of that site. *In vitro* studies have directly shown that, when Ca^2+^ levels fall, CaM unbinds from CaMKII, and that the rate at which this occurs is dramatically slowed if T286 is phosphorylated, a phenomenon called “trapping” [32]. Kinetic measurements indicate that, at Ca^2+^ concentrations similar to these in cells under basal conditions (~100 nM), the time constant of unbinding from phosphorylated CaMKII is ~10 sec, a value close to that of the fast component of decay of the WT Camui signal (tau1 ~6 sec). By comparison, *in vitro* studies showed that CaM unbinding from nonphosphorylated CaMKII is faster, occurring in ~1 sec [32]. This is similar to the 2 sec value reported for the 286A Camui mutant that cannot be autophosphorylated ([14] and S1 Fig). Of further relevance are biochemical studies *in vitro* showing that the autonomous state of the kinase (phosphorylated at T286 but without CaM bound), although much more active than when the enzyme is not phosphorylated, has only about 15–25% of the activity of the enzyme in the presence of Ca^2+^/CaM [8, 33]. This can be interpreted to mean that the regulatory sequence is only partially removed from catalytic sites by T286 phosphorylation, but is more fully removed by Ca^2+^/CaM. Based on these findings, one can entertain the following interpretation of the fast decay (tau1) of the Camui lifetime signal. During LTP induction, Ca^2+^/CaM binds to a large fraction of Camui, producing a large displacement of the regulatory and catalytic domain and thus a large increase in FRET lifetime. During this period, some or all WT Camui may become autophosphorylated on T286, leading to increased affinity to CaM (CaM trapping) [32]. The trapped CaM should allow for Camui to remain in an open state between repetitive synaptic stimulations, producing summation of the Camui FRET signal to low frequency stimuli, as observed for WT Camui. In phosphorylation deficient (T286A) Camui, however, CaM trapping cannot occur, and this limits efficient signal summation ([14] and S1 Fig). When, at the end of synaptic activation, Ca^2+^ falls, trapped CaM unbinds from autophosphorylated CaMKII in 5 ~10 sec or less ([32] [14] and Fig 1). This allows the regulatory region to approach the catalytic site and partially inhibit it. It seems plausible that it is this approach that is responsible for the fast component of the lifetime decay of Camui.

Although the ideas outlined above form a coherent description of some of the data, there is other data that point to additional complexity. First, measurements on purified Camui *in vitro* show that the fluorescence lifetime of autonomous Camui is large: it can be 70–80% [14, 15] and even 100% [31] of fully activated Camui in the presence of Ca^2+^/CaM. This is quite different from the ~20% magnitude of the slow tau2 component of Camui signal in live spines after LTP-inducing stimulus ([14] and Fig 1). One interpretation is that LTP-inducing stimuli produce only partial T286 autophosphorylation and therefore a fraction of the fast decay of Camui signal is due to release of CaM from nonphosphorylated CaMKII, which is faster (~1–2 sec rather than ~5–6 sec, see above). This explanation, however, has its own difficulties. First, the available data on WT Camui show no indication of this very fast (1 sec) component of decay, either after multiple uncaging stimuli or after a single nonsaturating stimulus [14]. Second, if the fraction of autophosphorylated kinase is small in spines, the fast decay of T286D/T305A/T306A Camui signal should be longer than the fast decay of WT but, in fact, it is shorter (Fig 2A and 2B).

A third complexity has to do with the nature of the slow component of the decline of the Camui signal. If the tau1 component of decay is due to unbinding of trapped CaM, then the following slow (tau2) component is expected to reflect behavior of autophosphorylated Camui. Consistent with this is our observation that the fractional magnitude of this slow component is increased by Calyculin A. However, it is puzzling that that slow component is present even in T286D/T305A/T306A and T286D/T305D/T306D Camui mutants, which cannot be either phosphorylated or dephosphorylated at T286 and, therefore, should not have the late component at all. These results seem to suggest that like the decay of the fast component, the decay of the slow component is also not directly related to the dephosphorylation of T286.

Each of these inconsistencies could have a reasonable explanation. For example, 286D/305A/306A mutation might not perfectly imitate T286 phosphorylation [34] and, therefore, might produce weaker CaM trapping, leading to the faster tau1 that is observed. It is also feasible that this mutant may be locked in a slightly larger open configuration after uncaging due to targeting or other interactions. Since binding of CaMKII to the NMDAR can lock the protein into an active (and thus presumably open) confirmation [35], binding reactions need to be considered when evaluating the basis of the slow component. In addition, at this time after glutamate uncaging, spine growth and diffusion of CaMKII out of spines become prominent [14, 36] which adds to the complexity of this phase of Camui signaling.

Another observation that requires discussion is that Calyculn A produced a dramatic decrease of the magnitude of the Camui response to uncaging, but only a very modest increase in the basal lifetime of Camui. This is different from the effect of the T286D/T305A/T306A mutant, which produced a relatively large increase in the basal lifetime but virtually no effect on the size of Camui response to uncaging. We have not investigated the process that caused the suppression of the Camui signal after Calyculin treatment. One possibility is that the suppression is a result of T305/T306 phosphorylation that inhibits CaM binding [9]. It is also possible that Calyculin A leads to a suppression of the Ca^2+^ rise during uncaging by regulating NMDA channels [37] or processes involved in Ca^2+^ homeostasis [38]. In any case, it seems clear that the suppression of the Camui response to uncaging is not a result of occlusion by the elevation of the basal lifetime because, as mentioned above, it was not observed in the T286D/T305A/T306A mutant or in experiments with expressible PP1 and PP2A inhibitors, which produced comparable increase in the basal lifetime but no change in the magnitude of response to uncaging.

One more noteworthy observation is that the T305A/T306A Camui mutant had a significantly larger basal lifetime than that of WT Camui, suggesting that the inhibitory phosphorylation of T305/T306 restricts Ca^2+^/CaM–dependent activation of WT Camui that may occur at basal conditions. Two recent observations are consistent with the notion of CaMKII activation by the basal Ca^2+^: Camui basal lifetime can be decreased by treating slices with the Ca^2+^/CaM competitive CaMK inhibitor, KN62 [14], or by reducing extracellular Ca^2+^ [24]. These basal effects of Ca^2+^/CaM apparently do not lead to autphosphorylation of T286 because the T286A Camui mutant has basal lifetime similar to that of WT Camui ([14], [24], Fig 2E). Altogether, these data suggest that under the basal conditions, CaMKII is partially activated by basal cytoplasmic Ca^2+^ without a significant contribution of the T286 autophosphorylated state, but restricted by partial inhibitory T305/T306 phosphorylation.

Considering this complex interpretation of Camui state under basal condition, it is not surprising that the increase in basal lifetime produced by Calyculin A is smaller than that produced by T286D/T305A/T306A mutations, which imitate 100% autophosphorylated state, but can be further activated by Ca^2+^/CaM without a restriction of inhibitory T305/T306 phosphorylations.

We conclude that interpretation of Camui dynamics during LTP induction is complicated. Additional studies implementing faster FRET recording, additional mutational analysis, and FRET calibration would help to clarify this complexity. Nevertheless, independent of any specific interpretation of each component of this signal, our results strongly suggest that neither the fast nor the slow components of decay of the Camui signal after LTP induction can be simply related to T286 dephosphorylation.

## Supporting Information

S1 Fig Preventing T286 autophosphorylation decreases the magnitude of the Camui response to glutamate uncaging and significantly shortens the decay of the response. Lifetime response to glutamate uncaging (7 pulses at 0.5 Hz) of WT Camui (filled symbols) and T286A Camui (open symbols). Circles–spine response. Squares–dendritic response. Green and brown lines—double exponential fits. Insert shows scaled single exponentials fits for WT (dash green, tau1 = 4.3 sec) and T286A (dash brown, tau1 = 2.1 sec) Camui respectively. The imaging period in these experiments was faster (1/4 sec, due to fewer sequential averaging performed during acquisition) than in all other experiments (1/8 sec, see Methods).
(TIFF)

S2 Fig Fast Camui deactivation is not affected by inhibitors of PP1, PP2A, and PP2B. (A–E) Graphs of WT Camui fluorescent lifetime change after glutamate uncaging under control conditions (filled symbols) and after treatment with protein phosphatase inhibitors. (A) Expression of PP1 protein inhibitor, mCherryPhi1(T57D)—Phi1(D), or (C) its variant, mCherryPhi1(T57A)—Phi1(A). (B) Expression of PP2A inhibitor, mCherrySET(S9D/S93D)-SET(D) or (D) its variant mCherrySET(S9A/S93A)—SET(A). (E) Treatment with PP2B inhibitor, FK506 (40 μM). All these treatments (A- E) produced no significant effect on the fast decay rate of Camui Glutamate uncaging protocol (eight pulses at 0.5 Hz, horizontal black bar) started at time 0. (F) Bar diagram showing change of basal fluorescence lifetime of Camui at conditions indicated in (A—E). Shadow line at the bottom indicates SE of basal lifetime for WT Camui. Stars indicate a statistically significant increase of the basal fluorescence lifetime in experimental conditions versus WT Camui. There was also statistically significant difference between basal lifetimes of Phi1(A) and Phi1(D) variants of the PP1 inhibitor (indicated by #), consistent with the increases of the inhibitory potency of the inhibitor by T57 phosphorylation (see S2 Text).
(TIF)

S3 Fig Expression patterns of PP1 inhibitor, Phi1(D), and PP2A inhibitor, SET(D). (A) Images of mCherry-tagged PP1 inhibitor, Phi1(D) (red), and co-expressed WT Camui (green) and green/red channel overlap showing that the inhibitor is strongly expressed in both cytoplasm and nucleus. (B) Images of mCherry-tagged PP2A inhibitor, SET(D) (red), and co-expressed WT Camui (green) and their overlap showing that this inhibitor is mostly expressed in nucleus. Expression patterns of less active variant of inhibitors of PP1, Phi1(A), and PP2A, SET(A) were in general similar to that of their more active counterparts (not shown). (C) Bar diagram showing cytoplasm/nucleus ratio of SET(A) and SET(D) expression estimated by their fluorescence intensity indicating that SET(D) variant had larger expression in the cytoplasm than SET(A) consistent with previous data (see S2 Text).
(TIFF)

S1 Text (DOCX)

S2 Text (DOCX)
