# Supplementary figures and images for: Fast Decay of CaMKII FRET Sensor Signal in Spines after LTP Induction Is Not Due to Its Dephosphorylation
Source: PLoS One. 2015 Jun 18;10(6):e0130457. doi: 10.1371/journal.pone.0130457 (PMC4472229; doi:10.1371/journal.pone.0130457)

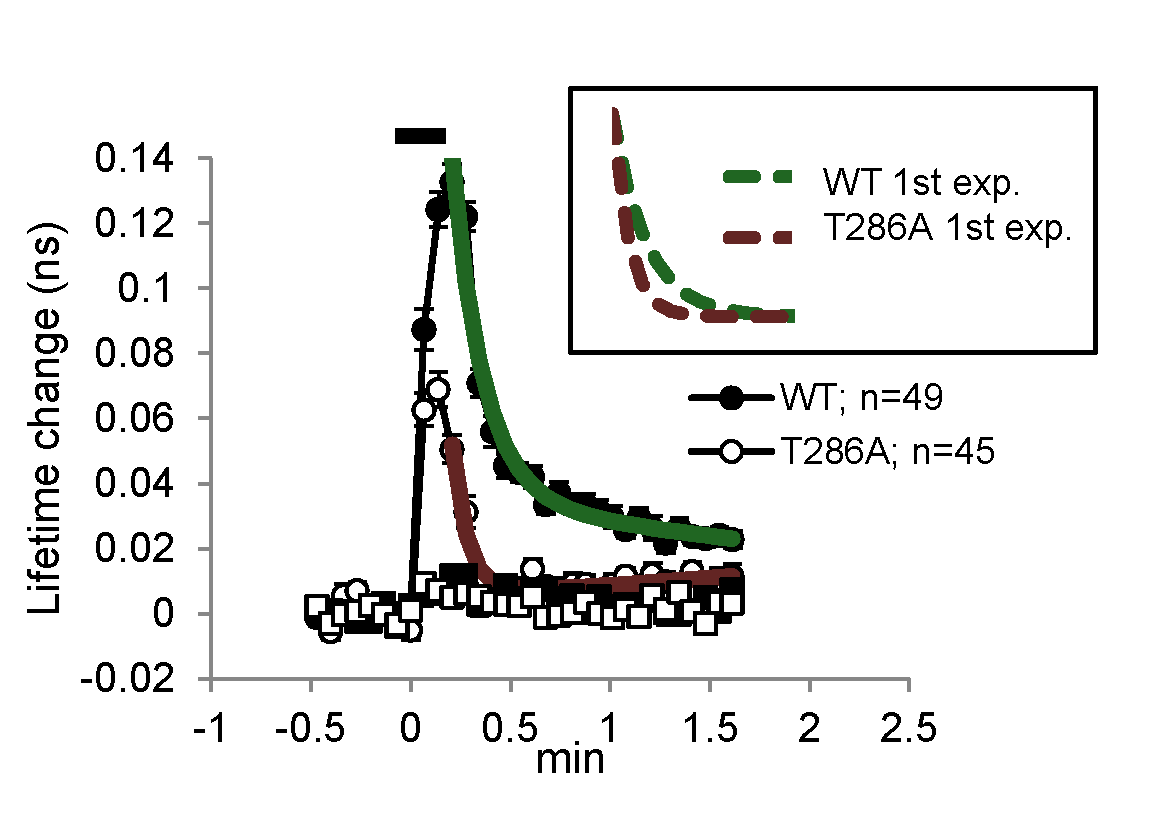

Supplement: S1 Fig — Lifetime response to glutamate uncaging (7 pulses at 0.5 Hz) of WT Camui (filled symbols) and T286A Camui (open symbols). Circles–spine response. Squares–dendritic response. Green and brown lines—double exponential fits. Insert shows scaled single exponentials fits for WT (dash green, tau1 = 4.3 sec) and T286A (dash brown, tau1 = 2.1 sec) Camui respectively. The imaging period in these experiments was faster (1/4 sec, due to fewer sequential averaging performed during acquisition) than in all other experiments (1/8 sec, see Methods). (TIFF) [file pone.0130457.s001.tiff]

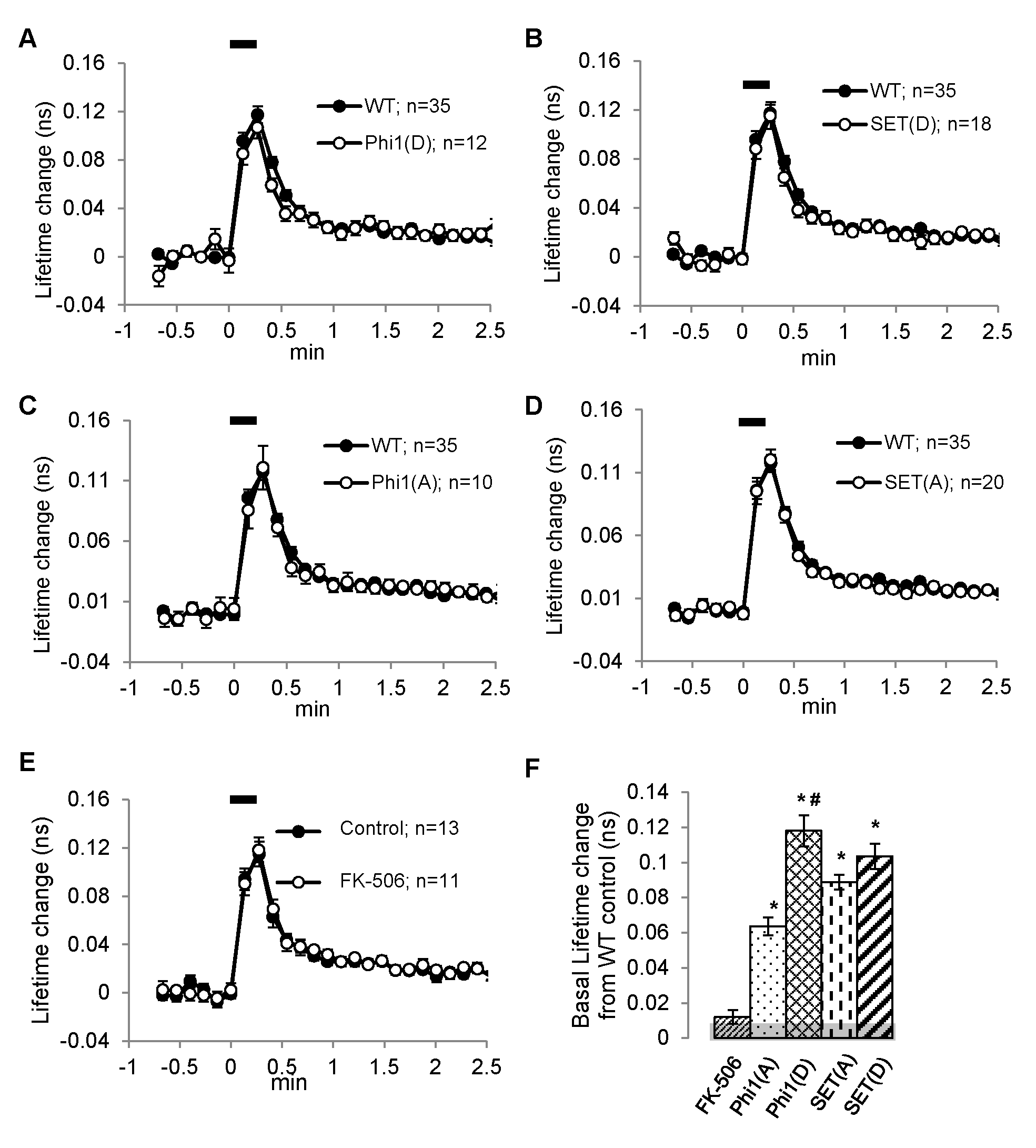

Supplement: S2 Fig — (A–E) Graphs of WT Camui fluorescent lifetime change after glutamate uncaging under control conditions (filled symbols) and after treatment with protein phosphatase inhibitors. (A) Expression of PP1 protein inhibitor, mCherryPhi1(T57D)—Phi1(D), or (C) its variant, mCherryPhi1(T57A)—Phi1(A). (B) Expression of PP2A inhibitor, mCherrySET(S9D/S93D)-SET(D) or (D) its variant mCherrySET(S9A/S93A)—SET(A). (E) Treatment with PP2B inhibitor, FK506 (40 μM). All these treatments (A- E) produced no significant effect on the fast decay rate of Camui Glutamate uncaging protocol (eight pulses at 0.5 Hz, horizontal black bar) started at time 0. (F) Bar diagram showing change of basal fluorescence lifetime of Camui at conditions indicated in (A—E). Shadow line at the bottom indicates SE of basal lifetime for WT Camui. Stars indicate a statistically significant increase of the basal fluorescence lifetime in experimental conditions versus WT Camui. There was also statistically significant difference between basal lifetimes of Phi1(A) and Phi1(D) variants of the PP1 inhibitor (indicated by #), consistent with the increases of the inhibitory potency of the inhibitor by T57 phosphorylation (see S2 Text). (TIF) [file pone.0130457.s002.tif]

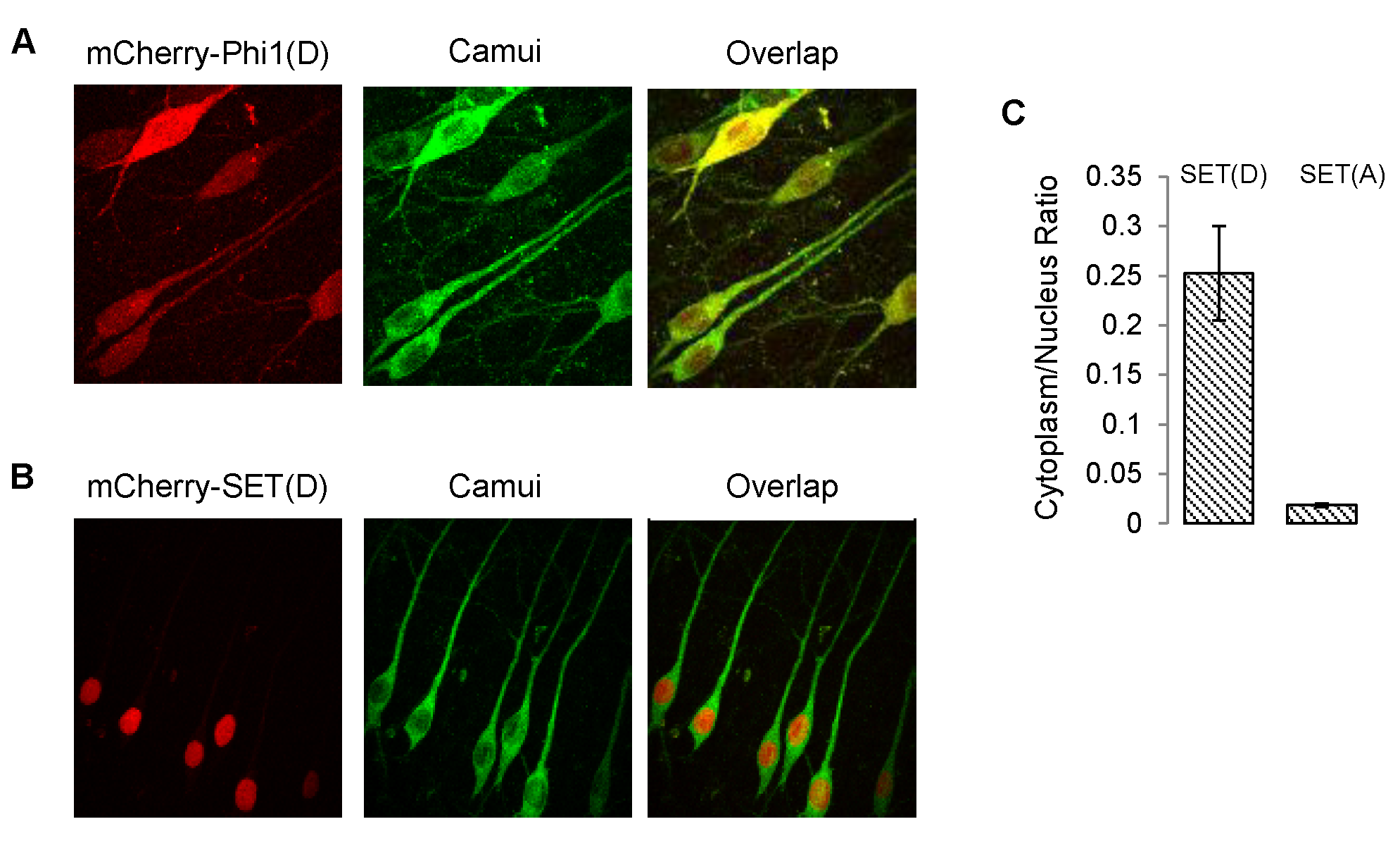

Supplement: S3 Fig — (A) Images of mCherry-tagged PP1 inhibitor, Phi1(D) (red), and co-expressed WT Camui (green) and green/red channel overlap showing that the inhibitor is strongly expressed in both cytoplasm and nucleus. (B) Images of mCherry-tagged PP2A inhibitor, SET(D) (red), and co-expressed WT Camui (green) and their overlap showing that this inhibitor is mostly expressed in nucleus. Expression patterns of less active variant of inhibitors of PP1, Phi1(A), and PP2A, SET(A) were in general similar to that of their more active counterparts (not shown). (C) Bar diagram showing cytoplasm/nucleus ratio of SET(A) and SET(D) expression estimated by their fluorescence intensity indicating that SET(D) variant had larger expression in the cytoplasm than SET(A) consistent with previous data (see S2 Text). (TIFF) [file pone.0130457.s003.tiff]
