# Supplementary material for: Fast Decay of CaMKII FRET Sensor Signal in Spines after LTP Induction Is Not Due to Its Dephosphorylation
Source: PLoS One. 2015 Jun 18;10(6):e0130457. doi: 10.1371/journal.pone.0130457 (PMC4472229; doi:10.1371/journal.pone.0130457)
Supplement: S1 Text — (DOCX) [file pone.0130457.s004.docx]

**S1 Text.** Two methods were used to calculate lifetime decay rates of response to glutamate uncaging. In the first method, the data of all individual experiments were averaged together and then fit with two exponentials. In the second method, the data from each individual uncaging experiment were first fitted with two exponentials and then averages were calculated. In this second method, only data with statistically significant goodness of the fit were included in the average. As a result, the final values of tau1 and tau2 were slightly but not dramatically different between the two methods. For example, for WT, Camui tau1was 5.8 sec, 76% (first method) versus 6.6 ± 0.7 sec, 64 ± 3% (second method); tau2: 97 sec, 24% (first method) versus 92 ± 24 sec, 36 ± 3% (second method). The difference is probably because responses of individual spines were significantly noisier than the averaged responses. In the text, only values obtained using the second method are presented. After some treatments, or with some Camui mutants, the accurate calculation of tau2 was not possible (the calculations gave negative tau2 values) because of short interval of recording. Therefore, instead of tau2 values and corresponding A2 percentage, we calculated the magnitude of the “slow component” as an average value between 1.5 and 2.5 min after glutamate uncaging.
