# Supplementary material for: Fast Decay of CaMKII FRET Sensor Signal in Spines after LTP Induction Is Not Due to Its Dephosphorylation
Source: PLoS One. 2015 Jun 18;10(6):e0130457. doi: 10.1371/journal.pone.0130457 (PMC4472229; doi:10.1371/journal.pone.0130457)
Supplement: S2 Text — (DOCX) [file pone.0130457.s005.docx]

**S2 Text.** Phi1(T57A) has been shown to have inhibitory potency for PP1 ~ 100 nm similar to native Phi1(T57) protein, but the phosphorylation of this site increases the potency ~ 50 fold [[1](#_ENREF_1)]. The aspartate substitution of T57 has not been tested *in vitro,* to our knowledge, and might have weaker potency. Therefore, our data showing that both PP1 inhibitors, Phi1(A) and Phi1(D), significantly increased the basal fluorescence lifetime of Camui but the Phi1(D) effect was larger than that of Phi1(A), consistent with their inhibitory potencies. SET inhibitor proteins are mostly localized in nucleus but its cytosolic expression increases upon phosphorylation of S9 residue [[2](#_ENREF_2),[3](#_ENREF_3),[4](#_ENREF_4)]. Phosphorylation of S9 and S93 also increases binding of SET to PP2A [[2](#_ENREF_2),[5](#_ENREF_5)] and facilitates inhibition of PP2A [[5](#_ENREF_5)]). Consistent with these data, we found that the cytoplasmic concentration of the SET(D) mutant with S9D/S93D substitutions was increased in comparison to SET(A) mutant with S9A/S93A substitutions. Since the Ki of apparently not phosphorylated native SET is ~ 2 -25 nM [[6](#_ENREF_6),[7](#_ENREF_7)], it was not surprising that both SET(A) and SET(D) inhibitors produced similar effect on the fluorescent lifetime of Camui. This is because the expression level of proteins in our experiments is in the range of tens of micromolars [[8](#_ENREF_8)].

Our results showing that PP2B inhibitor FK506 did not affect the deactivation rate of Camui (S2 Fig. E) is consistent with previously published data of [[9](#_ENREF_9)].

1. Eto M, Karginov A, Brautigan DL (1999) A novel phosphoprotein inhibitor of protein type-1 phosphatase holoenzymes. Biochemistry 38: 16952-16957.

2. ten Klooster JP, Leeuwen I, Scheres N, Anthony EC, Hordijk PL (2007) Rac1-induced cell migration requires membrane recruitment of the nuclear oncogene SET. EMBO J 26: 336-345.

3. Trakhtenberg EF, Wang Y, Morkin MI, Fernandez SG, Mlacker GM, et al. (2014) Regulating Set-beta's Subcellular Localization Toggles Its Function between Inhibiting and Promoting Axon Growth and Regeneration. J Neurosci 34: 7361-7374.

4. Yu G, Yan T, Feng Y, Liu X, Xia Y, et al. (2013) Ser9 phosphorylation causes cytoplasmic detention of I2PP2A/SET in Alzheimer disease. Neurobiol Aging 34: 1748-1758.

5. Vasudevan NT, Mohan ML, Gupta MK, Hussain AK, Naga Prasad SV (2011) Inhibition of protein phosphatase 2A activity by PI3Kgamma regulates beta-adrenergic receptor function. Mol Cell 41: 636-648.

6. Li M, Guo H, Damuni Z (1995) Purification and characterization of two potent heat-stable protein inhibitors of protein phosphatase 2A from bovine kidney. Biochemistry 34: 1988-1996.

7. Li M, Makkinje A, Damuni Z (1996) The myeloid leukemia-associated protein SET is a potent inhibitor of protein phosphatase 2A. J Biol Chem 271: 11059-11062.

8. Otmakhov N, Lisman J (2012) Measuring CaMKII concentration in dendritic spines. J Neurosci Methods 203: 106-114.

9. Fujii H, Inoue M, Okuno H, Sano Y, Takemoto-Kimura S, et al. (2013) Nonlinear decoding and asymmetric representation of neuronal input information by CaMKIIalpha and calcineurin. Cell Rep 3: 978-987.
